# Potential Therapeutic Effect of ZnO/CuO Nanocomposite as an Acaricidal, Immunostimulant, and Antioxidant in Rabbits

**DOI:** 10.3390/vetsci12040333

**Published:** 2025-04-04

**Authors:** Shimaa R. Masoud, Said I. Fathalla, Sherif M. Shawky, Hanem El-Gendy, Mahboba A. Z. Alakhras, Rashed A. Alhotan, Anam Ayyoub, Shaimaa Selim, Khaled Defallah Al-Otaibi, Ahmed M. A. El-Seidy

**Affiliations:** 1 Physiology Department, University of Sadat City, Sadat City 32897, Egypt; shaimaa.masoud@vet.usc.edu.eg (S.R.M.); shsh00076@vet.usc.edu.eg (S.M.S.); mahbouba.abelwanees.p2016@vet.usc.edu.eg (M.A.Z.A.); 2 Department of Pharmacology, University of Sadat City, Sadat City 32897, Egypt; hanem.elgendy@vet.usc.edu.eg; 3 Department of Animal Production, College of Food and Agriculture Sciences, King Saud University, Riyadh 11451, Saudi Arabia; ralhotan@ksu.edu.sa; 4 College of Life Sciences, Northwest A & F University, Yangling District, Xianyang 712100, China; anamayyoub@nwafu.edu.cn; 5 Department of Nutrition and Clinical Nutrition, Faculty of Veterinary Medicine, Menoufia University, Shibin El-Kom 32514, Egypt; 6 Department of Psychology, Faculty of Arts, Cairo University, Cairo 12613, Egypt; 10512020105378@stud.cu.edu.eg; 7 Inorganic Chemistry Department, Advanced Materials Technology and Mineral Resources Research Institute, National Research Centre, 33 El-Bohouth St., Dokki, Cairo 12622, Egypt; am.elseidy@nrc.sci.eg

**Keywords:** ZnO/CuO nanocomposites, immunity, *S. scabies* mites, rabbits, ivermectin

## Abstract

The present study aimed to identify a safe and novel approach using zinc oxide/copper oxide nanocomposites (AZ) to enhance growth parameters, immunity, and fight Sarcoptic mange in vitro and in vivo in rabbits. In vitro: the acaricidal activity of AZ was assessed at concentrations of AZ-25: 2.5% *w*/*w* AZ/molasses, AZ-125: 12.5% *w*/*w* AZ/molasses, and controls (normal saline, molasses, and Ivermectin) every hour for seven hours under a stereoscopic microscope. In vivo: involved 40 rabbits (10 replicates/group). G1 served as the control negative group (normal un-infected rabbits), G2 served as the control negative group (infected rabbits), the animals in the G3 group were given a combination of AZ (40 mg/kg body weight (BW)) and molasses (5 mg/mL), and G4 served as the control to the vehicle; receiving molasses 8 mL/kg BW twice weekly for 6 weeks. Blood, serum, and tissue samples were collected at the middle and the end of the trial. AZ was made using the sonication sol–gel method. X-ray diffraction (XRD) and X-ray photoelectron spectroscopy (XPS) were performed to confirm the crystal structure, purity, particle size, and oxidation states. AZ showed immunostimulant, acaricidal, and antioxidant effects with normal tissue histological structure and low tissue residual levels. Additionally, there were improvements in blood interferon-gamma, immunoglobulin (Ig) M, IgG, phagocytic activity, phagocytic index, globulin, and total protein in the AZ group. The XRD patterns of AZ were coordinated by XRD reference codes Crystallography Open Database (COD): 9016326 for Tenorite (CuO) and by XRD reference COD: 9004179 for Zincite (ZnO). The CuO and ZnO crystal sizes were 21.87 Å and 24.89 Å, respectively. The XPS spectra indicated the presence of Cu as Cu (II) and Zn as ZnO.OH and ZnO. In conclusion, AZ exhibited antioxidant, acaricidal, and immunostimulant effects, with mild residues in the brain, liver, and kidney tissues, while maintaining a normal histological structure of tissues.

## Simple Summary

Currently, nanotechnology is more widely applied with the inclusion of innovative instruments for consuming nanoparticle-sized critical elements, boosting the animal’s ability to absorb these components, and therefore optimizing their performance. The present study aimed to study the effects of zinc oxide/copper oxide nanocomposites (AZ) in vitro and in vivo to enhance growth parameters, immunity, and fight Sarcoptic mange in rabbits. Our findings revealed that AZ can be used as an antioxidant, acaricidal, and immunostimulant, with mild residues in the brain, liver, and kidney tissues, while maintaining a normal histological structure of tissues in rabbits.

## 1. Introduction

Rabbits are one of the most popular livestock species because of their ability to produce meat and their economic, reproductive, and productive efficiency. Meat from rabbits is regarded as an essential source of protein for humans due to its inordinate quality, minimal fat content, and low cholesterol content. To take full advantage of the manufacturing of rabbit meat, there is a significant demand for essential nutritional supplements to improve the growth performance of developing rabbits [1]. The current study, along with previous studies, utilized trace elements and their nanocomposites to enhance the immunity and growth performance in rabbits. Recently, zinc nutrition has grown into a huge task at the head of many researchers’ minds. Zinc is essential for immune function, wound healing, protein and DNA synthesis, growth, hormone secretion, and the metabolism of other enzymes [2,3]. The concentration of Zn in fresh nutritional materials is inadequate to fulfill the requirements of the animals. Furthermore, it cannot be stored in an animal’s body, thus there is a need for frequent replenishment of Zn in the animals’ diets. In addition, rabbits’ diets have high phytate content that lowers Zn absorption [4]. Currently, nanotechnology is more widely applied with the inclusion of innovative instruments for consuming nanoparticle-sized critical elements, boosting the animal’s ability to absorb these components, and therefore optimizing their performance. Zinc oxide nanoparticles (ZnONPs) can improve productive performance by acting as antibacterial mediators, boosting immunity, and enhancing rabbit growth and reproduction [5]. Using nano-ZnO exhibited superior results and is less harmful than traditional Zn sources; therefore, lowering the required quantity [6]. ZnO-NPs induced wound healing and decreased the apparent signs of infection, hastening reepithelization [7]. Furthermore, copper (Cu) is one of the key micronutrients and has a crucial role in the production of metalloproteins by acting as a cofactor in the directive of enzyme activity [8]. Cu is involved in boosting the immune system to combat infections and Cu deficiency often results in increased susceptibility of hosts to infection [9]. Inorganic Cu and organic Cu sources had different effects on cecal microbiota composition in rabbits, which might be valuable to the lower occurrence of diarrhea and increased growth performance in rabbits [10]. Copper oxide nanoparticles (CuONPs) have received attention and are commonly used in feeds for high bioavailability, referring to small size particles [11]. Studies show that CuONPs enter through the endocytosis pathway [12]. Furthermore, CuONPs have a high dissolution capacity, can relieve free Cu ions, and are transported through Cu transporters [13]. CuONPs induced greater growth performance and a better metabolic rate in broilers and rabbits, respectively, [14,15]. CuONPs have gained increased interest because of their inherent qualities that are not detectable in viable Cu such as optical, catalytic, electrical, antiparasitic, antibacterial, and antifungal [16]. Furthermore, it has been found that the bimetallic nanoparticles CuO/ZnO NPs have substantial antibacterial, cytotoxic, and antioxidant properties, suggesting their potential in a variety of therapeutic treatments [17]. The biological synergistic effects of ZnO/CuO NPs decreased ROS generation, enhanced apoptosis of cancer cells, and Cu genotoxicity. These elements can be used as effective therapeutic agents to treat lung cancer and melanoma. Because ROS can induce DNA damage, ZnO-NPs can be used extensively as antioxidants [18].

The objective of the current trial was to examine the potential immunomodulatory and growth-promoting properties of ZnO/CuO nanocomposite, and therefore the application of CuO-NPs and ZnO-NPs in combination against *S. scabiei* mites. Therefore, the acaricidal activity of these compounds was assessed both in vitro and in vivo in rabbits that were experimentally infected with *S. scabiei* mites.

## 2. Materials and Methods

### 2.1. Materials

Sigma Aldrich (USA) provided the starting ingredients, which were HPLC analytical grade or higher.

### 2.2. Synthesis of AZ

AZ was synthesized using the sonication sol–gel method [19,20]. PEG (1.5 g) was added to the KOH solution followed by basic copper carbonate (4.77 g CuCO_3_.Cu(OH)_2_.H_2_O (21.57 mmol)) under sonication (2 min, 20 cycles, 2 min pause). Zinc acetate monohydrate (6.20 g Zn (Zn (CH_3_COO)_2_.H_2_O) (28.25 mmol), 120 mL) solution was added to the reaction mixture under sonication (2 min, 15 cycles, 2 min pause). The mixture was stirred at 1000 rpm at 85 °C. The fluid was dried before it was crushed into granules. The powders were crushed after being calcined at 650 °C for 5 h in an air-filled furnace. The powder was rinsed with DD several times and dried overnight at 100 °C.

### 2.3. Experimental Schemes

#### 2.3.1. Nanocomposite

X-ray diffraction was used to evaluate the sample’s structure and crystallite size. The X-ray diffraction (XRD) patterns were produced using an X’pert PRO diffractometer with a Cu-radiation (λ = 1.542Å) at 45 K.V. and 35 mA over a range of 2θ = 2–60°. The average size of the crystallites was determined using the Debye–Scherrer equation. X-ray photoelectron spectroscopy (XPS) was collected on K-ALPHA (Thermo Fisher Scientific, Waltham, Massachusetts, USA) with monochromatic X-ray Al K-alpha radiation −10 to 1350 e.v, spot size 400 μm at a pressure 10^−9^ mbar with full spectrum pass energy 200 e.v, and at narrow spectrum 50 e.v.

#### 2.3.2. Experimental Animals

For this investigation, 40 white male New Zealand rabbits, aged 30–35 days (shortly after weaning), with body weights (BW) of 600 ± 200 g from a farm located in Badder Centre (Beheira, Egypt) were used. Following their assignment to experiments, male rabbits were kept in their cages for one week for acclimatization. One group of rabbits was healthy and examined clinically and housed individually in wire cages, containing feeding hoppers and automatic drinkers, and the other three groups of artificially infested rabbits were used in the trial. In a dedicated room with sufficient ventilation, a temperature range of 35 ± 5 °C, and a humidity level of 45 ± 10%, each group kept ten male rabbits to enhance stress and decrease immunity. At the end of the study, each rabbit was fed 120 g of chow per day, beginning with 50 g and increasing weekly [21]. Animal handling was carried out following the recommendations and regulations of the Animal Care House. All surgeries were conducted under anesthesia (isoflurane anesthetic drug) to minimize animal discomfort. After placing each rabbit in an induction anesthetic box, a piece of cotton with anesthesia was inserted inside the box.

### 2.4. Growth Performance Measurement

The daily feed intake was measured on a replication basis. After a 12 h fast, the live body weight of each rabbit was measured weekly to determine the average daily feed intake (ADFI), average daily gain (ADG), and feed intake: weight gain (FC) ratio on a duplicate basis.

### 2.5. Body Weights and Spleen Somatic Index

Rabbits included in the experiment were weighed using a weight measurement scale before the beginning of treatment and on the scarification day. Upon being sacrificed by cervical dislocation, the spleen was carefully removed, cleaned from any extraneous tissue, and weighed. Spleen somatic index was calculated as follows [22]:

Spleen somatic index = [Absolute spleen weight (g)/body weight of rat (g)] × 100.

### 2.6. Induction of Infection

The mite-infested skin crusts were conveyed to the ear canals of healthy rabbits (free from mites). To keep the crusts from coming off, pieces of cotton were crusted with crusts and then inserted into the rabbit’s ear. After one to two hours, these pieces of cotton were removed [23].

#### Parasites

**Figure 1:**
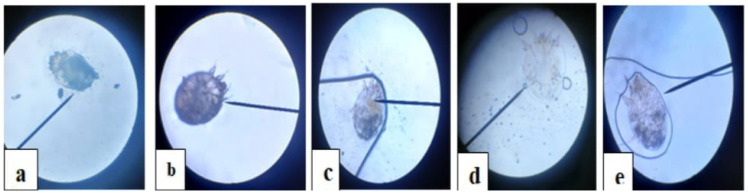
Dorsal aspects of *S. scabiei* mites under the light microscope. (Convex dorsally) (**a**) control, (**b**) AZ-20, (**c**) molasses, (**d**) AZ-10, and (**e**) iv-SD.

Two naturally infected rabbits with *S. scabiei* were used as a source of *S. scabiei* mites to induce crusted scabies. Scabies mite infection in rabbits was detected by scraping the borders of skin sores with a scalpel blade till the capillary blood became visible. To enhance mite transfer to the dish surface, recovered scales were placed in Petri plates and incubated for 30 min at 30 °C [24,25]. The plates were examined using a light microscope (Olympus Optical Co., Ltd., Tokyo, Japan) to identify the morphological features of the *S. scabiei* mites, as shown in Figure 1.

### 2.7. Experimental Protocol

#### 2.7.1. In Vitro Assay

**Figure 2:**
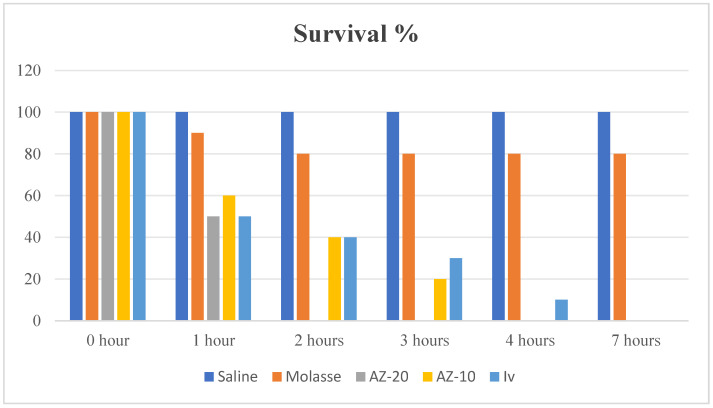
The percentage of viable adult mites.

To investigate the AZ effect on mite survival, 20 adult live mites (10/Petri dishes) were subject to molasse (AZ-20: 19.84 μg/mg, AZ-10: 9.92 μg/mg), respectively. For comparison, 30 adult live mites (10/Petri dishes) were subject to molasse, saline, and IV solution (50 μg/mL), respectively. Under a stereoscopic microscope (Olympus SZX7, Evident Europe GmbH, Hamburg, DE), the number of viable 65 mites in each group was counted hourly for seven hours, as shown in Figure 2. Once the mites were lightly touched with a probe, mortality was measured by searching for movement. Additionally, as shown in Figure 1, one dead mite was removed from each group for examination under a light microscope.

#### 2.7.2. In Vivo Assay

This research was performed according to the ethical standards approved by the Institutional Animal Care and Use Committee of the Faculty of Veterinary Medicine, University of Sadat City, Egypt (Ethical approval number: VUSC-032-1-24; approved on 29.5.2024). All experimental procedures were performed following the ethical guidelines approved by the National Institutes of Health for the Use and Treatment of Laboratory Animals. A total of 40 male New Zealand rabbits were randomly allocated into four treatment groups, with ten rabbits per group (10 replicates/group). The control group was fed a basal diet and served as the control negative group (normal non-infected rabbits, G1), the infected group (G2) was infected with *S. scabiei* and was fed the basal diet, the G3 group was given a combination of AZ and molasses (M-AZ, 5 mg/mL, 5 mg AZ = 2.43 mg Cu (2.92 mg CuO) + 1.58 mg Zn (2.08 mg ZnO), the dosage was 8 mL/kg BW of M-AZ orally (40 mg/kg BW AZ) twice a week for 6 weeks, in addition to the basal diet, and the G4 group, acted as a vehicle, received molasses 8 mL/kg BW in addition to the basal diet. It should be mentioned that to meet the nutritional requirements of rabbits, the basal diet contained 50 mg/kg Zn and 4 mg/kg Cu. This trial lasted for 6 weeks. All rabbits were provided with feed and water ad libitum. The rabbit’s diet was composed of soybean meal (44% CP), sunflower cake (28% CP), wheat bran, barley, yellow corn, clover hay, wheat straw, common salt, dicalcium phosphate, premix, and L-lysine. The chemical composition of the diet is presented in Table 1.

### 2.8. Samples

#### 2.8.1. Blood Sample

Blood was drawn from the ear vein using vacuum tubes at the halfway point of the experiment (after 3 weeks) and at the end of the trial (after 6 weeks) at the same time of day. This blood sample was divided into two parts: the first part was collected on EDTA (whole blood) and used for determining hematological parameters (WBC count and differential leukocyte count) using a Unit 3060 automatic cell analyzer (Automated Hematology Analyzer, Frankfurt am Main, Germany), while the other part was left to coagulate at room temperature for 20 min, then refrigerated for clot and centrifuged at 3000 rpm for 15 min. The clean supernatant serum was collected and frozen at −20 °C for biochemical analysis. Sreelakshmi et al. [26] described a method for performing phagocytosis of neutrophils using Candida albicans, total protein Biomed’s total protein kit (EGY-CHEM for lab technology, Badr City, Egypt; catalog No. TP116250) and the ERBA device, albumin Diamond’s albumin kit (EGY-CHEM for lab technology, Badr City, Egypt; catalog No. BK-467858D) and the ERBA device, and globulin calculated according to Buzanovskii [27].

#### 2.8.2. Tissue Samples

Kidney, liver, and spleen tissue samples were preserved in 10% neutral buffered formalin, and further examined under a light microscope for histopathological analysis. Additional tissue samples were obtained from the brain, muscles, liver, and for determining the AZ residues in tissues. After processing each sample per the standard procedure [28], and the AZ residual was estimated.

### 2.9. Antioxidants Profile

Samples of sera were gathered to measure antioxidant markers such as superoxide dismutase (SOD, catalog No. CSB-EL022397RA) and malondialdehyde (MDA, catalog No. MBS268427) according to Rashed et al. [29]. SOD and MDA were analyzed using SOD and MDA Elisa kits (CUSABIO Co., Houston, TX 77054, USA) following the instructions guidelines.

### 2.10. Serum Immunity Profile

Samples of sera were gathered for this investigation. In compliance with Chen et al. [30], the interferon-gamma, immunoglobulin (Ig) M, IgG, phagocytic activity (PAC), and Phagocytic Index were measured according to Sreelakshmi et al. [26]. An analysis was performed for the albumin, globulin, and total protein concentrations [27].

### 2.11. AZ Residuals

The tissue residuals of Zn and Cu were determined using the Baraud et al. method [31] using Agilent 5100 Inductively Coupled Plasma Optical Emission Spectrometer (ICP-OES) Kits (Merck Company, Darmstadt, Germany) with Synchronous Vertical Dual View (SVDV). The analytical wavelengths and equipment detection limitations are as follows: Cu 324.752 nm; DL 1 μg·L^−1^; Zn 206.200 nm; DL 10 μg·L^−1^. An agate mortar was used to melt and then homogenize the material. 5.0 mL of 65% HNO_3_ (SuprapurTM, Merck KGaA, Darmstadt, Germany) and 1 mL of 30% H_2_O_2_ (SuprapurTM, Merck KGaA, Darmstadt, Germany) were introduced to quartz pressure vessels containing weighed samples (1 g). The sealed containers were put in a mineralizer with temperature and pressure controls. Emissions of radiation from the prepared solutions were measured. Zn and Cu contents were expressed in ppm.

### 2.12. Histopathological Examination

Tissue samples (kidney, Liver, and spleen) were wholly fixed in 10% neutral buffered formalin for examination using a light microscope, all samples were pounded briefly in tap water, dehydrated in ascending grades of ethyl alcohol, cleared in methyl benzoate or xylene and embedded in paraffin wax. The paraffin-embedded tissue samples were cut into 4–6 µm sections using a Leica rotatory microtome (Leica Microsystems, Wetzlar, Germany), and finally, they were stained by hematoxylin and eosin stain (H & E, catalog No. CY 1577) (scale bar = 50 µm) [32].

### 2.13. Statistical Analysis

Before statistical analysis, the Shapiro test was used to ensure the data values were normally distributed. For the data, the means ± S.E. are shown. The data were assessed using the Tukey–Kramer Post hoc test following one-way ANOVA. The significance level for each statistical test was set at *p* < 0.05 [33].

## 3. Results and Discussion

### 3.1. XRD Characterization

**Figure 3:**
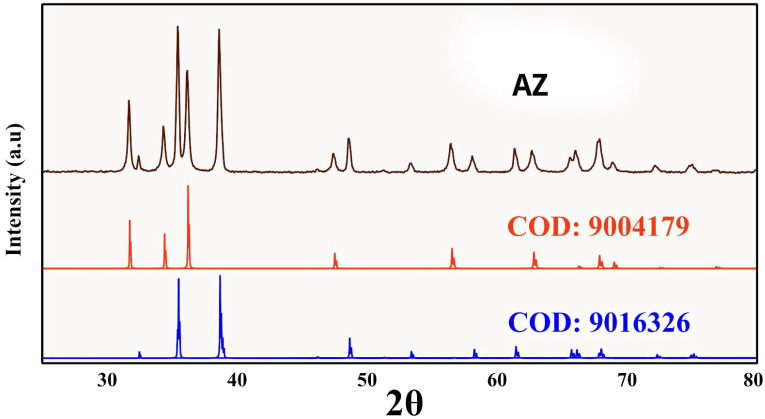
XRD spectra of AZ.

The sharp and well-defined peaks are shown in Figure 3, which indicates the good crystallinity of the nanocomposites. XRD spectra were harmonized with structure files retrieved from COD (Crystallography Open Database). The XRD patterns of AZ were matched by XRD reference codes COD: 9016326 for Tenorite (CuO) and by XRD reference COD: 9004179 for Zincite (ZnO). The diffraction peaks at 2θ = 32.42°, 35.34°, 35.45°, 38.59°, 38.80°, 46.13°, 48.59°, 51.19°, 53.34°, 56.56°, 58.07°, 61.36°, 65.61°, 66.01°, 66.24°, 67.67°, 67.88°, 68.68°, 71.45°, 72.12°, 72.71°, 74.75°, 75.01°, and 79.48° regions are indexed to (1 1 0), (0 0 2), (1 1 −1), (1 1 1), (2 0 0), (1 1 −2), (2 0 −2), (1 1 2), (0 2 0), (0 2 −1), (2 0 2), (1 1 −3), (0 2 2), (3 1 −1), (3 1 0), (1 1 3), (2 2 0), (2 2 −1), (3 1 −2), (3 1 1), (2 2 1), (0 0 4), (2 2 −2), and (0 2 −3), respectively, for the monoclinic crystal system of Tenorite with space group C 1 c 1 (9). The diffraction peaks at 31.68°, 34.33°, 36.15°, 47.40°, 56.42°, 62.67°, 66.17°, 67.74°, 68.87°, 72.35°, and 76.71° are indexed to (1 0 0), (0 0 2), (1 0 1), (1 0 −2), (1 1 0), (1 0 −3), (2 0 0), (1 1 2), (1 1 −2), (0 0 4), and (202), respectively, for the hexagonal system of Zincite with space group P 63 m c (186). Scherrer’s formula using FWHM (full-width half maximum) was used to calculate the crystallite sizes of CuO, and ZnO, see Table 1. The crystallite sizes and structural parameters of CuO, and ZnO were calculated using the most intense peak in each phase, see Table 2. The most intense peak for CuO was 35.45° for plane (1 1 −1). Peaks at 31.68° (1 0 0), 34.33° (0 0 2), and 36.15° (1 0 1) were used to calculate a and c atomic parameters (c/a = 1.60) for ZnO phase. Inter-planar spacing for planes (1 0 0), (0 0 2), and (1 0 1) were 2.82, 2.61, and 2.48 Å, respectively. Axial ratio, unit cell volume, oxygen position parameter, zinc oxygen bond length L were 16.02 × 10^−1^, 48.01 Å, 37.99 × 10^−2^, and 19.57 × 10^−2^.

### 3.2. XPS

**Figure 4:**
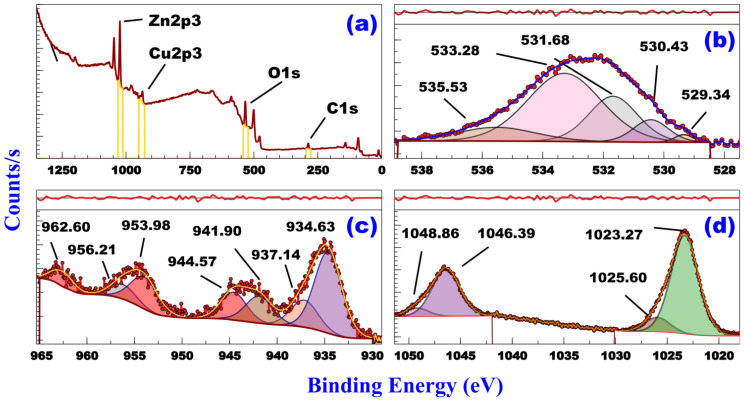
XPS spectra of AZ. (**a**) survey, (**b**) O-1s, (**c**) Cu-2p, and (**d**) Zn-2p XPS spectra of AZ.

The XPS survey spectrum of AZ is shown in Figure 4a. It indicates the presence of Zn-2p, Cu-2p, O-1s, and a small trace of C-1s. The presence of C may be caused by the adsorption on the sample surface or during sample preparation for analysis. The fitted curves are in agreement with the experimental curves in all high-resolution spectra. Figure 4b shows the XPS high-resolution spectrum (HRs) of O1s. The asymmetric figure can be resolved into five components by Gaussian fitting. The spectrum shows multiple peaks in the 529.34–530.43 eV range, which can be attributed to the presence of metal oxides [34,35,36]. The spectrum of AZ shows peaks at 535.53 eV and 533.28 eV, which may be attributed to Oγ associated with water species and oxygen in surface OH species, respectively, [37,38]. Figure 4c shows the deconvoluted Cu-2p XP spectrum of AZ. The peaks at 934.63 eV and 953.98 eV regions are attributed to Cu-2p_3/2_ and Cu-2p_1/2_, respectively, in CuO [39,40]. AS shows peaks associated with Cu^2+^ state in CuO (941.90–944.57 eV and 956.21 eV), a characteristic peak of Cu^2+^ ions (937.14 eV) and a peak indicating an unfilled Cu 3d^9^ shell (962.60 eV) which supports the existence of Cu^2+^ in the sample [41,42,43]. The absence of any peaks below 934 eV indicates that Cu^+^ was absent [41,44]. Figure 4d shows the deconvoluted Zn-2p XP spectrum of AS. This spectrum indicates the presence of multiple Zn-2p_3/2_ and Zn-2p_1/2_ peaks in the 1046.39–1048.86 eV and 1023.27–1025.60 eV regions, respectively, with a spin–orbits splitting value of ≈23 eV. These characteristic peaks indicate the presence of Zn as ZnO (Zn-2p_1/2_: 1046.39 eV and Zn-2p_3/2_: 1023.27) and ZnO.OH (Zn-2p_1/2_: 1048.86 eV and Zn-2p_3/2_: 1025.60 eV) [19]. The average concentration ratio of ZnO.OH/ZnO (15.49%) is determined depending on the following equation:

$$\frac{ZnO.OH}{ZnO}=\frac{\sum ZnO.OH\;peak\;area(2p_{3/2}+2p_{1/2})\times 100}{\sum of\;ZnO+ZnO.OH\;peaks\;areas}$$

### 3.3. In Vitro Assessment

The acaricidal effect of AZ against *S. scabiei* was detected in the current inquiry with IV as the standard drug (Figure 2). Two concentrations of AZ (19.84 (AZ-20) and 9.92 (AZ-10) μg/mL) and one concentration (50 μg/mL) of IV were used. Figure 2 shows the percentage of viable adult mites treated with saline, AZ-20, AZ-10, molasse, and IV. AZ-20 and IV showed the lowest viable mites after one h (50%) with a maximum death of 50% followed by AZ-10 (60%). After 2 h, AZ-20 was able to cause the complete death of all mites (0% viable mites) followed by AZ-10 and IV (40%). To achieve this result, AZ-10 needed 4 h. The standard drug (IV) needed 7 h. According to Simonart and Lam Hoai [45], after a long period of IV use as an acaricide, scabies mites survival duration was long, this may have been attributed to improved Multidrug Resistance-Associated Proteins (MDRP) expression, which are particles related to the resistance to some drugs, including IV. To the best of our knowledge, no previous report has determined the effect of AZ composite on *S. scabiei*. Instead, Zn and Cu were used independently in previous studies. Moreover, AZ was used in a lower concentration (19.84 and 9.92 μg/mL) compared with IV (50 μg/mL), and it was more effective against *S. scabiei*. The mechanism by which ZnO-NPs deactivate the parasite is poorly understood [46]. ZnO-NPs in the current study induced antiparasitic action. ZnO-NPs can break down the glycoproteins that make up the oocyst wall of the parasite, which allows them to penetrate more easily and increases the amount of dangerous Zn^2+^ ions, as demonstrated previously [47]. Furthermore, ZnO-NPs’ photosensitive properties were triggered by light, as reported by [48]. This led to the production of ROS, which have an impact on the parasite virulence-associated surface glycoprotein molecules. Furthermore, the NPs’ small size made it simple to slip through cell membrane pores and have a direct harmful effect by interfering the vital metabolic functions [49]. Cu^2+^ can create ROS through various chemical reactions, which can damage DNA strands and change how genes are expressed [50]. Nano CuO can directly interfere with essential metabolic processes by slipping through cell membrane holes [51].

### 3.4. In Vivo Assessment

**Figure 5:**
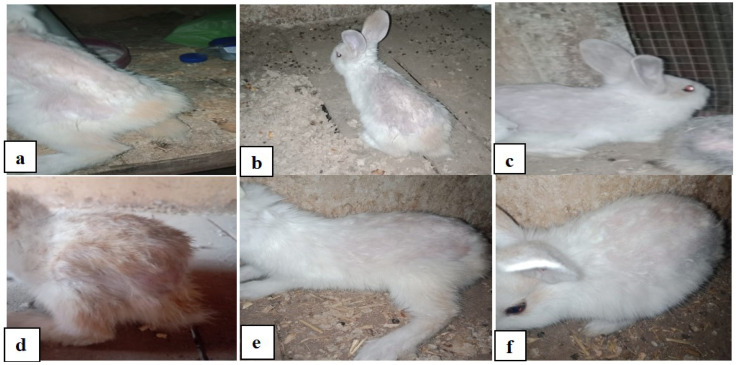
(**a**) Rabbits treated with AZ during the 1st week: the infected rabbit, showing the appearance of mite lesion over the back of rabbit including crusts, dandruff, exudation, and alopecia; (**b**) 2nd week: the infected rabbit stopping of erythema, fewer crusts, and alopecia -continuing treatment with AZ; (**c**) 3rd week: the infected rabbit stopping of erythema, no crusts, and rabbit’s fur starts to grow—continuing treatment with AZ; (**d**) 4th week: the infected rabbit showing a noticeable healing of skin lesion with longer hair; (**e**) 5th week: rabbit showing almost worthy healing of skin lesion with longer hair—continuing treatment with AZ; (**f**) 6th week rabbit showing complete healing of skin lesion with healthy fur at the last week of experiment for animals treated with AZ.

AZ enhanced the immune system, and the therapeutic effect of AZ on *S. scabiei* -infected rabbits was observed. When compared to IV, AZ induced better antiparasitic activity. The animals received AZ have the ability to cure parasite illness and boost immunity. As seen in Figure 5, these animals consequently became more resistant to the parasitic illness and tolerant to high temperatures and humidity.

### 3.5. Effect of AZ on the Growth Parameters, Leucogram, Immunity Profile, and Some Biochemical Parameters

#### 3.5.1. Effect of Nanocomposite of AZ on Growth Performance and Spleen Somatic Index of the Rabbits

Rabbits in G1 appeared healthy, while the other groups had S. mange infection. These rabbits appeared diseased with low performance and bad health conditions. There was non-significant mortality occurring during the experiment. A mortality rate was recorded during the experiment in each group and calculated as a percentage (%) as shown in Table 3.

Table 4 shows the effects of AZ on BW, feed intake, feed conversion, weekly body weight gain, and spleen somatic index of rabbits. Non-significant differences in the spleen somatic index were observed among the treatment groups. The results showed that the administration of AZ to infected animals improved the BW of rabbits during the experimental periods (*p* < 0.05). The results showed that supplementing rabbits with AZ significantly reduced feed intake and improved feed conversion (*p* < 0.05). The results in Table 3 showed that the total BW gains of rabbits supplemented with AZ were significantly greater than those of the rabbits in the G1 and G2 groups (*p* < 0.05). These results were similar to the findings of Abdel-Wareth et al. [4] and Moustafa et al. [52] who reported that dietary supplementation of ZnONPs enhanced BW, weight gain, and feed efficacy. In addition, CuONPs were also reported to improve growth parameters and increase weight and weight gain in New Zealand rabbits [15].

#### 3.5.2. The Effect of Nanocomposite of AZ on Blood WBCs, Neutrophils, Lymphocytes, Monocytes, and Eosinophils Counts of the Rabbits

The data presented in Table 5 revealed that total WBCs in G3 were significantly increased (*p* < 0.05) than in the G1, G2, and G4 groups at both the mid and end of the experiment. In addition, the G3 rabbits had higher neutrophils and lymphocyte % (*p* < 0.05) than the G1, G2, and G4 ones at mid and end of the experiment. The findings of the current study are in agreement with Effah-Yeboah et al. [53] who demonstrated that there was an elevation in the hematological parameters such as the red blood cell count, hemoglobin, and WBCs due to Cu and Zn supplements. According to Mahmoud et al. [54], a decrease in the WBC count was observed due to Zn supplementation in fish which disagreed with the current study. On the other hand, Refaie et al. [55] revealed that there were non-significant differences in WBC levels due to nano Cu supplementation, but there is a high percentage of lymphocytes. Changes in Zn levels act as a stimulus that interferes with specific and non-specific immunity, as well as Zn was reported to augment granulocyte recruitment and phagocytosis [56].

#### 3.5.3. Effect of Nano Composite of AZ on Immunity Profile (Interferon Gamma, IgG, IgM, Phagocytic Activity and Phagocytic Index) of the Rabbits

The data shown in Table 6 indicated that G3 and G4 groups had greater interferon-gamma, IgM, IgG, phagocytic activity %, and phagocytic index (*p* < 0.05) than G1 and G2. Additionally, Compared to G1 and G2, G3 exhibited higher phagocytic activity percentage and phagocytic index. The current study’s results were consistent with those of El-Moghazy et al. [1] and Cui et al. [57], who found that animals supplemented with zinc had significantly higher levels of immunoglobulins such as IgG, IgM, and IgA than the non-supplemented group. Additionally, in line with our findings, Chand et al. [58] and Bartlett and Smith [59] observed that groups fed dietary zinc had higher levels of IgM and IgG than the control. According to Mahmoud et al. [54], zinc oxide had no effect on IgG, IgM, or interferon-gamma, which was contrary to the findings of the present investigation. Because zinc is essential for polynucleotide transcription and the process of genetic expression, it has a significant impact on cellular and humoral immunity, which is why zinc supplementation results in higher levels of immunoglobulins [60]. Copper administration is the cause of enhanced immunity because it enhances energy production, which impacts the immune system, boosts ceruloplasmin production, and promotes immune cell differentiation [61].

#### 3.5.4. The Effect of AZ on Total Protein, Albumin, and Globulin of Rabbit

The data shown in Table 7 revealed that the blood total protein levels of the G3 group followed by the G4 group were significantly increased (*p* < 0.05) than those in the G2 group at the middle of the experiment, but G2 had higher blood total protein concentration (*p* < 0.05) than the G4 group in the end of the experiment. Albumin showed a non-significant difference (*p* > 0.05) between the treatment groups at the middle of the experiment; however, G2 showed a significant increase (*p* < 0.05) in albumin concentration than G3, and G4 at the end of the experiment. Globulin concentration in the G3 group was significantly higher (*p* < 0.05) than those in the G1, G2, and G3 groups at the middle and end of the experiment. The A/G ratio was significantly increased in the G2 rabbits (*p* < 0.05) compared to the G3 ones at the middle and end of the experiment. The current study does not align with the findings of Badawi et al. [62], Wang et al. [63], and Cho et al. [64], who found no impact of zinc supplementation on either albumin or globulin. The current study’s results were consistent with those of Oconitrillo et al. [65], who demonstrated that zinc supplementation raised several immunoglobulins, including IgG, IgM, and IgA, compared to the control group. According to Kumar et al. [66], Cu-NPs enhance fish globulin and total protein. Mohammed et al. [67] showed that ZnO-NPS in broilers did not affect the total protein and albumin compared to the control, which contradicts our findings but is consistent with ZnO-NPS increased globulin and decreased A/G ratio. Zinc is necessary for several enzymes that aid in protein synthesis and production, which explains why globulin levels rise while consuming zinc supplements [68]. According to research by AL-Ruwad et al. [69], increased globulin with Cu-NPs supplementation is related to enhanced ceruloplasmin production since copper is a rate-limiting factor for ceruloplasmin synthesis (90% of copper is attached to ceruloplasmin protein).

#### 3.5.5. Effect of AZ on the Antioxidant Profile of the Rabbits

The data shown in Table 8 revealed that the SOD of G3 was significantly increased (*p* < 0.05) when compared to G1, G2, and G4 at the end of the experiment. On the other hand, the MDA of G3 was significantly lower in the G2 group and there was non-significant difference in MDA among the G1, G2, and G4. Zinc supplementation increases SOD because zinc is needed to synthesize antioxidant enzymes, and SOD scavenges excess ROS (inhibits their creation) by increasing their transcription [70]. Additionally, the mechanism of ZnO-NPs as an antioxidant refers to the fact that zinc is a well-known antioxidant metal, a crucial component of antioxidant enzymes such as SOD, and a known sulfhydryl group protector. It is also thought to prevent lipid peroxidation by displacing transition metals such as iron and copper from catalytic sites. ZnO-NPs can lower MDA levels, increase antioxidant enzyme activity, and shield cell membrane integrity from oxidative stress damage. It can decrease levels of free radicals, increase antioxidant activity, and improve antioxidant activities [71].

#### 3.5.6. AZ Residues in the Liver, Back Muscle, and Brain of Rabbits

The data shown in Table 9 revealed that the Zn and Cu residue in the liver tissue was higher in the G3 group (*p* < 0.05) than in the G1, G2, and G4 groups. In the back muscle, Zn and Cu residues of the G3 group were significantly greater (*p* < 0.05) when compared to those of the G1, G2, and G4 groups. In addition, Zn and Cu residues in the brain of G3 were significantly greater (*p* < 0.05) than in the G1, G2, and G4 groups. Increased Cu residue in the liver is due to the liver being the target organ for Cu and because food intake determines the amount of Cu in the liver, which is also easily retained and generate residues in the liver [72]. Because the liver is the primary organ for excreting copper [73], copper builds up in the liver until excretion is finished. This is the cause of the buildup of copper in hepatic tissue. Compared to muscle, the liver had a greater zinc retention rate [74]. Improving intestinal zinc absorption and hepatic sequestration may significantly increase the amount of zinc in the liver [75].

#### 3.5.7. AZ Histopathological Pictures of Rabbits’ Kidney, Liver, and Spleen

**Figure 6:**
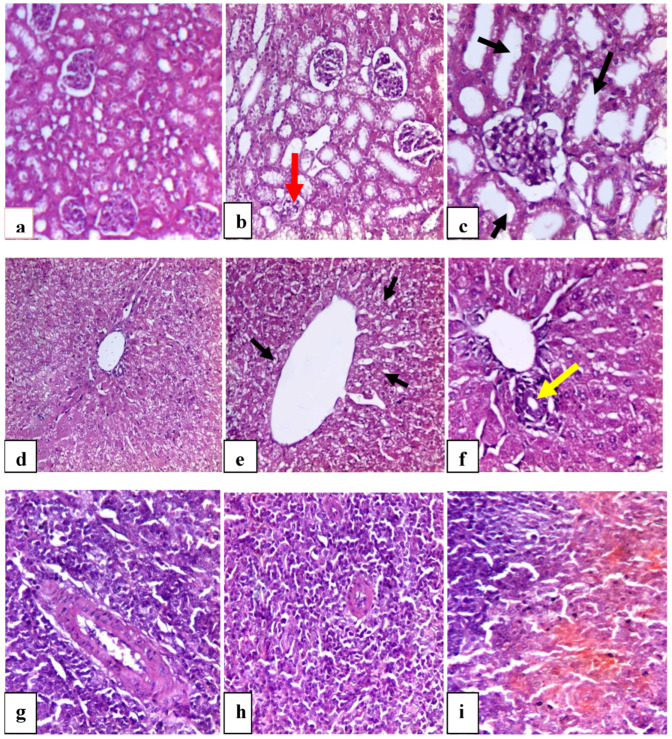
A photomicrograph showing the renal histopathological structure. (**a**) Control normal kidney tissue (X20). (**b**) AZ treated group. (**c**) Molasses-treated group (X20). Hepatic histopathological structure (X20). (**d**) Control normal liver tissue (X10). (**e**) AZ treated group (X20). (**f**) Molasses-treated group (X20). Splenic histopathological structure. (**g**) Control normal spleen tissue (X20). (**h**) AZ treated group (X20). (**i**) Molasses-treated group (X20). Scale bar 50 µm. Red arrow shows atrophy of the glomeruli, black arrow in (**c**) shows completely normal renal tubules, black arrow in (**e**) shows mild vacuolar degeneration of the hepatocytes near the central vein, and yellow arrow show newly formed bile duct near the central vein.

Figure 6 illustrates the effect of treated groups on liver, kidney, and spleen histopathological morphology (H-Es20). Figure 6a shows the normal histological structure of the renal cortex. The renal cortex is the outer layer of kidney tissue. Bowman’s capsule surrounds the glomerulus with typical Bowman’s space in the renal corpuscles. Figure 6b displayed a normal histological structure, except for a small region of the renal cortex that showed glomerular atrophy (red arrow). The renal corpuscle, renal tubules, and interstitial tissue were all normal. Figure 6c shows a normal histological structure. Interstitial tissue, the renal corpuscle, and the proximal and distal convoluted tubules were all normal; nevertheless, some of the tubules were swollen (black arrow). Figure 6d shows a normal hepatic tissue architecture. The central vein is lined by a single layer of endothelial cells and plates of hepatocytes radiate outward from the central vein towards the portal triad. The liver parenchyma is composed of small hexagonal lobules with portal passages at the apexes. The hepatocytes are organized inside the lobules as cell cords that connect the peripheral portal tracts to the central veins. Hepatocyte has abundant granular eosinophilic cytoplasm, basophilic centrally located round to ovoid nuclei, and prominent nucleoli, and are separated by sinusoids which are spaces lined by fenestrated endothelial cells and contain macrophages called Kupffer cells. Figure 6e shows a normal histological picture of the liver tissue with mild vacuolar degeneration of the hepatocytes close to the central vein. Figure 6f displayed a newly formed bile duct close to the central vein (yellow arrow), revealing that the hepatocytes, sinusoids, and central veins in the molasses-consuming animals were all in normal condition. Figure 6g shows a normal appearance, with red pulp making up most of the splenic parenchyma and white pulp (lymphocytes) encircling a central arteriole. Most of the stromal splenic tissue is made up of the red pulp. The splenic cords and splenic sinusoids make up this structure. The reticular connective tissue holds the splenic cords together. The cords are made up of groups of cells that contain blood, lymphocytes, and macrophages. To make up the white pulp, there are central arterioles and well-defined areas of B lymphocytes. These are the lymphoid follicles of the spleen. Splenic sinusoids, which are located between the splenic cords and filled with blood, give the white pulp its distinctive white appearance. Figure 6h showed a normal spleen structure. The red and white pulps are clearly identified with the central arterioles. Figure 6i shows a normal spleen structure. The red and white pulp is evident with central arterioles compared to the control group. The data gathered verified that the histological structure of the liver, kidney, and spleen was normal in all treatment groups. This reveals that oral administration of ZNO-NPS did not cause alteration in the histological structures of these tissues, which is in line with Abd Elmonem et al. [76] who reported that ZnONPs ameliorate the effect of irradiation in albino rats. The histological examination showed that low concentrations of ZnO/CuO core/shell nanoparticles are safe for desired histological and biomedical requests [77,78].

## 4. Conclusions

The current findings revealed that ZnO/CuO nanocomposite can be used in a lower concentration (19.84 and 9.92 g/mL) as an acaricidal agent. AZ-20 and AZ-10 showed encouraging in vitro acaricidal effectiveness against *S. scabiei* mites. This effect was further confirmed with an in vivo trial. AZ supplementation maintained normal tissue histological structure while exhibiting immunostimulant and antioxidant properties. Mild remnants were also found in the back muscles, liver, and brain. It was concluded that ZnO/CuO nanocomposite administration had immunostimulant, antioxidant, acaricidal, and growth-promoting effects on rabbits,

## Figures and Tables

**Table 1:** Chemical composition of the diet.

Chemical Composition	Ration
Dry matter	90.7%
Crude protein	18%
Digestible energy	2600 Kcal/kg
Crude fiber	16%
Calcium	1.2%
Phosphorus	0.8%
Lysine	0.75%
Methionine and cysteine	1.2%
Zinc	50 mg/kg
Copper	4 mg/kg

**Table 2:** Structural and atomic parameters.

	Zincite (ZnO)	Tenorite (CuO)
	COD: 9004179	COD: 9016326
a *	3.26 (3.25)	4.70 (4.69)
b *		3.43 (3.43)
c *	5.22 (5.21)	5.15(5.14)
2 θ *	36.15°	35.45°
FWHM	0.40	0.35
Crystallite size (nm)	21.87	24.89
Microstrain (ϵ × 10^−3^)	5.33	4.78
Specific surface area (S m^2^g^−1^)	48.44	37.19
Lorentz factor (Lf)	2.73	2.83
Lorentz polarization factor	18.05	18.85

* (COD value), and a, b, and c in Å.

**Table 3:** Effect of AZ on the mortality rate of experimentally infected rabbits with *S. scabiei* (n = 10/group).

Groups	Dead Rabbit/Total Rabbits	Mortality (%)
G1	0/10	0%
G2	2/10	20%
G3	0/10	0%
G4	1/10	10%

Values in the same column are presented as means ± SE (n = 10 per group). Values with different superscripts are significantly different at *p* < 0.05. G1, the control negative group (normal un-infected rabbits); G2, the control negative group (infected rabbits); G3, the animals in this group were given a combination of AZ (5 mg/mL of molasses) and the dosage was 40 mg/kg BW of AZ; and G4 served as the control group, acted as a vehicle, it received molasses 8 mL/kg BW.

**Table 4:** Effect of nanocomposite of AZ on growth performance and spleen somatic index of the rabbits.

Weeks	Parameters	Treatments			
		G1	G2	G3	G4
	Initial BW (gm)	800 ± 11.0	805 ± 11.04	793 ± 9.14	810 ± 13.15
W1	BW (gm)	1100 ± 2.22 ^b^	1009 ± 23.71 ^b^	1291 ± 24.68 ^a^	962 ± 19.57 ^b^
	WG (gm)	300 ± 15.65 ^ab^	204 ± 18.11 ^b^	497 ± 30.11 ^a^	151 ± 20.99 ^b^
	FI (gm)	440 ± 0.002 ^b^	474 ± 0.001 ^a^	425 ± 0.76 ^c^	438 ± 0.66 ^b^
	FC	1.46 ± 0.54 ^b^	2.42 ± 0.26 ^a^	0.86 ± 0.04 ^c^	3.14 ± 0.37 ^a^
W2	BW	1250 ± 10.54 ^b^	1091 ± 30.21 ^c^	1468 ± 9.60 ^a^	1276 ± 29.42 ^b^
	WG	150 ± 10.55 ^b^	82.16 ± 9.79 ^c^	176 ± 29.12 ^b^	314 ± 19.95 ^a^
	FI	520 ± 0.76 ^a^	506 ± 0.76 ^b^	506 ± 0.76 ^b^	532 ± 0.76 ^a^
	FC	3.46 ± 0.67 ^b^	6.70 ± 0.97 ^a^	3.49 ± 0.81 ^b^	1.73 ± 0.11 ^b^
W3	BW	1390 ± 6.25 ^b^	1216 ± 5.62 ^c^	1693 ± 6.15 ^a^	1414 ± 6.02 ^b^
	WG	140 ± 8.53 ^b^	124 ± 10.24 ^b^	224 ± 10.83 ^a^	137 ± 25.76 ^b^
	FI	530 ± 0.76 ^c^	670 ± 0.76 ^b^	582 ± 0.76 ^c^	689 ± 0.76 ^a^
	FC	3.78 ± 0.57 ^b^	5.56 ± 0.46 ^ab^	2.61.12 ^b^	6.59 ± 1.74 ^a^
W4	BW	1590 ± 3.52 ^b^	1398 ± 9.45 ^c^	1809 ± 9.69 ^a^	1636 ± 21.37 ^b^
	WG	200 ± 6.5 ^ab^	181 ± 30.99 ^ab^	116 ± 10.44 ^b^	221 ± 20.42 ^a^
	FI	685 ± 0.76 ^c^	752 ± 0.76 ^b^	704 ± 0.76 ^c^	769 ± 0.76 ^a^
	FC	3.43 ± 1.25	6.19 ± 2.54	6.31 ± 0.60	3.63 ± 0.35
W5	BW	1850 ± 3.25 ^b^	1580 ± 17.52 ^c^	2017 ± 4.23 ^a^	1856 ± 23.79 ^b^
	WG	260 ± 3.05	181 ± 10.15	208 ± 8.08	220 ± 23.53
	FI	690 ± 0.76 ^b^	737 ± 0.76 ^b^	612 ± 0.76 ^c^	767 ± 0.76 ^a^
	FC	2.65 ± 0.23 ^b^	4.11 ± 0.22 ^a^	2.96 ± 0.12 ^b^	3.70 ± 0.41 ^ab^
W6	BW	1985 ± 1.52 ^b^	1799 ± 9.28 ^c^	2243 ± 3.47 ^a^	2001 ± 5.04 ^b^
	WG	135 ± 3.22 ^b^	219 ± 12.56 ^a^	225 ± 5.35 ^a^	145 ± 25.30 ^b^
	FI	700 ± 0.76 ^b^	747 ± 0.76 ^a^	690 ± 0.76 ^b^	749 ± 0.76 ^a^
	FC	5.18 ± 0.20 ^a^	3.45 ± 0.17 ^b^	3.07 ± 0.07 ^b^	5.83 ± 0.81 ^a^
Spleen weight (g) SSI		1.95 ± 0.13	2.00 ± 0.06	2.00 ± 0.08	1.80 ± 0.02
		0.101 ± 0.04	0.11 ± 0.04	0.09 ± 0.06	0.09 ± 0.06

In the same row, values are presented as means ± SE (n = 10 per group). Values with different superscripts are significantly different at *p* < 0.05. G1, the control negative group (normal un-infected rabbits); G2, the control negative group (infected rabbits); G3, the animals in this group were given a combination of AZ (5 mg/mL of molasses) and the dosage was 40 mg/kg BW of AZ; and G4 served as the control group, acted as a vehicle, it received molasses 8 mL/kg BW. BW, body weight; WG, weight gain; FI, feed intake; FC, feed conversion; SSI, spleen somatic index.

**Table 5:** The effect of AZ on blood total white blood cell (WBC) and differential leukocytic count.

Parameters	Time	Treatments			
		G1	G2	G3	G4
White blood cells (10^3^/mm^3^)	Mid of Exp.	6.50 ± 0.96 ^b^	6.42 ± 0.96 ^b^	10.32 ± 1.05 ^a^	5.90 ± 0.41 ^b^
	End of Exp.	6.08 ± 0.26 ^b^	6.00 ± 0.26 ^b^	7.52 ± 0.15 ^a^	5.12 ± 0.24 ^c^
Neutrophil (%)	Mid of Exp.	29.20 ± 1.25 ^b^	31.20 ± 1.25 ^b^	45.00 ± 2.02 ^a^	41.20 ± 1.06 ^a^
	End of Exp.	32.83 ± 0.98 ^b^	33.83 ± 0.98 ^b^	43.16 ± 2.67 ^a^	28.83 ± 2.02 ^b^
Lymphocyte (%)	Mid of Exp.	51.60 ± 2.58 ^b^	49.60 ± 2.58 ^b^	55.60 ± 1.12 ^a^	50.20 ± 1.70 ^b^
	End of Exp.	52.03 ± 0.60 ^b^	50.03 ± 0.60 ^b^	52.86 ± 1.83 ^a^	46.86 ± 2.05 ^b^
Monocytes%	Mid of Exp.	3.50 ± 0.71 ^a^	4.50 ± 0.71 ^a^	5.00 ± 0.44 ^a^	5.00 ± 0.51 ^a^
	End of Exp.	2.50 ± 0.22 ^a^	3.50 ± 0.22 ^a^	3.33 ± 0.33 ^a^	3.50 ± 0.42 ^a^
Eosinophils%	Mid of Exp.	1.22 ± 0.30 ^a^	1.83 ± 0.30 ^a^	2.00 ± 0.36 ^a^	1.50 ± 0.34 ^a^
	End of Exp.	1.30 ± 0.22 ^a^	1.50 ± 0.22 ^a^	1.16 ± 0.16 ^a^	1.16 ± 0.16 ^a^

In each row, the values are presented as means ± SE (n = 10 per group). Values with different superscripts are significantly different at *p* < 0.05. G1, the control negative group (normal un-infected rabbits); G2, the control negative group (infected rabbits); G3, the animals in this group were given a combination of AZ (5 mg/mL of molasses) and the dosage was 40 mg/kg BW of AZ; and G4 served as the control group, acted as a vehicle, it received molasses 8 mL/kg BW.

**Table 6:** The effect of AZ on the immunity profile of the rabbits.

Items	Treatments			
	G1	G2	G3	G4
Interferon gamma (pg/mL)	645 ± 2.06 ^c^	518.71 ± 2.06 ^c^	1186.2 ± 2.91 ^a^	736.34 ± 1.20 ^b^
IgM (pg/mL)	322 ± 14.25 ^c^	284 ± 14.25 ^c^	588 ± 21.86 ^a^	338 ± 4.40 ^b^
IgG (pg/mL)	175 ± 3.05 ^c^	169 ± 3.05 ^c^	557 ± 9.59 ^a^	258 ± 1.85 ^b^
Phagocytic activity, %	52 ± 2.006 ^b^	44.37 ± 2.006 ^b^	63.12 ± 1.511 ^a^	61.72 ± 1.85 ^a^
Phagocytic index	1.75 ± 0.064 ^b^	1.57 ± 0.064 ^b^	2.04 ± 0.143 ^a^	2.20 ± 0.041 ^a^

In each row, the values are presented as means ± SE (n = 10 per group). Values with different superscripts are significantly different at *p* < 0.05. G1, the control negative group (normal un-infected rabbits); G2, the control negative group (infected rabbits); G3, the animals in this group were given a combination of AZ (5 mg/mL of molasses) and the dosage was 40 mg/kg BW of AZ; and G4 served as the control group, acted as a vehicle, it received molasses 8 mL/kg BW.

**Table 7:** Effect of AZ on serum total protein, albumin, and globulin of the experimental rabbits.

Parameters	Time	Treatments			
		G1	G2	G3	G4
Total protein (g/dL)	Mid of Exp.	5.00 ± 0.17 ^ab^	4.55 ± 0.19 ^b^	5.78 ± 0.06 ^a^	5.46 ± 0.04 ^a^
	End of Exp.	5.50 ± 0.07 ^a^	5.50 ± 0.09 ^a^	5.67 ± 0.13 ^a^	5.21 ± 0.05 ^b^
Albumin (g/dL)	Mid of Exp.	3.20 ± 0.13 ^a^	3.034 ± 0.182 ^a^	3.33 ± 0.129 ^a^	3.40 ± 0.046 ^a^
	End of Exp.	3.25 ± 0.02 ^a^	3.27 ± 0.08 ^a^	3.05 ± 0.035 ^b^	3.09 ± 0.031 ^b^
Globulin (g/dL)	Mid of Exp.	1.80 ± 0.037 ^ab^	1.52 ± 0.037 ^c^	2.45 ± 0.08 ^a^	2.05 ± 0.04 ^b^
	End of Exp.	2.25 ± 0.012 ^b^	2.22 ± 0.012 ^b^	2.60 ± 0.07 ^a^	2.11 ± 0.05 ^b^
A/G Ratio	Mid of Exp.	1.77 ± 0.022 ^b^	1.99 ± 0.153 ^a^	1.35 ± 0.11081 ^b^	1.65 ± 0.058 ^b^
	End of Exp.	1.44 ± 0.039 ^a^	1.47 ± 0.039 ^a^	1.17 ± 0.031 ^b^	1.46 ± 0.051 ^a^

In each row, the values are presented as means ± SE (n = 10 per group). Values with different superscripts are significantly different at *p* < 0.05. G1, the control negative group (normal un-infected rabbits); G2, the control negative group (infected rabbits); G3, the animals in this group were given a combination of AZ (5 mg/mL of molasses) and the dosage was 40 mg/kg BW of AZ; and G4 served as the control group, acted as a vehicle, it received molasses 8 mL/kg BW. A/G ratio, albumin to globulin ratio.

**Table 8:** Effect of AZ on the antioxidant profile of the rabbits.

Items	Treatments			
	G1	G2	G3	G4
SOD, U/mL	100.00 ± 1.45^b^	70.00 ± 1.52^c^	139.00 ± 0.57^a^	118.00 ± 1.52^b^
MDA, nmol/mL	6.59 ± 0.28 ^b^	9.59 ± 0.25 ^a^	3.83 ± 0.17638 ^b^	5.1667 ± 0.60 ^b^

In each row, the values are presented as means ± SE (n = 10 per group). Values with different superscripts are significantly different at *p* < 0.05. G1, the control negative group (normal un-infected rabbits); G2, the control negative group (infected rabbits); G3, the animals in this group were given a combination of AZ (5 mg/mL of molasses) and the dosage was 40 mg/kg BW of AZ; and G4 served as the control group, acted as a vehicle, it received molasses 8 mL/kg BW. SOD, superoxide dismutase; MDA, malondialdehyde.

**Table 9:** AZ residues in the liver, back muscle, and brain of rabbits (ppm).

Item	Group	Tissue		
		Liver	Back muscle	Brain
Zinc	G1	97.88 ± 1.29 ^b^	58.98 ± 1.25 ^b^	40.88 ± 1.28 ^b^
	G2	98 ± 1.52 ^b^	60 ± 1.25 ^a^	40 ± 0.88 ^b^
	G3	155.25 ± 1.44 ^a^	62.00 ± 0.57 ^a^	50.27 ± 0.14 ^a^
	G4	100.00 ± 1.52 ^b^	59.50 ± 1.25 ^a^	42.66 ± 0.88 ^b^
Copper	G1	10.88 ± 0.29 ^b^	1.55 ± 0.29 ^b^	9.00 ± 0.19 ^b^
	G2	11.00 ± 0.29 ^b^	1.60 ± 0.06 ^b^	9.05 ± 0.09 ^b^
	G3	30.50 ± 0.28 ^a^	2.55 ± 0.14 ^a^	9.38 ± 0.09 ^a^
	G4	12.43 ± 0.29 ^b^	1.50 ± 0.06 ^b^	9.23 ± 0.07 ^b^

In each column, the values are presented as means ± SE (n = 10 per group). Values with different superscripts are significantly different at *p* < 0.05. G1, the control negative group (normal un-infected rabbits); G2, the control negative group (infected rabbits); G3, the animals in this group were given a combination of AZ (5 mg/mL of molasses) and the dosage was 40 mg/kg BW of AZ; and G4 served as the control group, acted as a vehicle, it received molasses 8 mL/kg BW.

## Data Availability

The data presented in this study is available on request from the corresponding author.
